# Seronegative patients vaccinated with cytomegalovirus gB-MF59 vaccine have evidence of neutralising antibody responses against gB early post-transplantation

**DOI:** 10.1016/j.ebiom.2019.11.005

**Published:** 2019-11-15

**Authors:** Ilona Baraniak, Ariane C. Gomes, Isabella Sodi, Toby Langstone, Emily Rothwell, Claire Atkinson, Sylvie Pichon, Fabienne Piras-Douce, Paul D. Griffiths, Matthew B. Reeves

**Affiliations:** a Institute for Immunity and Transplantation, UCL, Royal Free Campus, London NW3 2PF, United Kingdom; b Clinical Development, Sanofi Pasteur, Marcy l'Etoile, France; c Research and Non-Clinical Safety, Sanofi Pasteur, Marcy l'Etoile, France

**Keywords:** Cytomegalovirus, Vaccination, Antibody responses, Prime-boost

## Abstract

**Background:**

Human cytomegalovirus (HCMV) causes a ubiquitous infection which can pose a significant threat for immunocompromised individuals, such as those undergoing solid organ transplant (SOT). Arguably, the most successful vaccine studied to date is the recombinant glycoprotein-B (gB) with MF59 adjuvant which, in 3 Phase II trials, demonstrated 43–50% efficacy in preventing HCMV acquisition in seronegative healthy women or adolescents and reduction in virological parameters after SOT. However, the mechanism of vaccine protection in seronegative recipients remains undefined.

**Methods:**

We evaluated samples from the cohort of seronegative SOT patients enroled in the Phase II glycoprotein-B/MF59 vaccine trial who received organs from seropositive donors. Samples after SOT (0–90 days) were tested by real-time quantitative PCR for HCMV DNA. Anti-gB antibody levels were measured by ELISA. Neutralization was measured as a decrease in infectivity for fibroblast cell cultures revealed by expression of immediate-early antigens.

**Findings:**

Serological analyses revealed a more rapid increase in the humoral response against gB post transplant in vaccine recipients than in those randomised to receive placebo. Importantly, a number of patient sera displayed HCMV neutralising responses – neutralisation which was abrogated by pre-absorbing the sera with recombinant gB.

**Interpretation:**

We hypothesise that the vaccine primed the immune system of seronegative recipients which, when further challenged with virus at time of transplant, allowed the host to mount rapid immunological humoral responses even under conditions of T cell immune suppression during transplantation.

**Research in context**

Evidence before this study:

Our understanding of HCMV vaccine mediated protection in seronegative solid organ transplant recipients is limited. Attempts to identify protective immunological mechanisms have focused on characterisation of immune responses immediately following vaccination. The analyses so far have failed to provide evidence for protection being mediated by conventional humoral mechanisms such as neutralization or activation of antibody dependant cellular cytotoxicity.

Added value of this study:

We present, for the first time, analyses of the post-transplant immunological responses of vaccinated individuals after they had been challenged with the virus at the time of transplant. The results are consistent with the gB/MF59 vaccine priming the pre-transplant immune system of seronegative recipients because, upon challenge with the virus, vaccinees rapidly generated an elevated gB antibody response that included classical neutralizing activity. Importantly, the response was greater than that seen in recipients of placebo.

Implications of all the available evidence:

These studies highlight the importance of studying immune responses beyond the immediate post vaccination phase. In practice, solid organ transplantation provides a tractable human challenge model for HCMV and shows how pharmacodynamic assessment of candidate vaccines may potentially identify correlates of immune protection. We recommend that this extended study design is considered when novel HCMV vaccines are evaluated in the future with separate analyses of initial immune priming and immune response to subsequent viral challenge.

**CRediT authorship contribution statement**

**Ilona Baraniak:** Data curation, Formal analysis, Writing - original draft. **Ariane C. Gomes:** Data curation, Formal analysis, Writing - original draft. **Isabella Sodi:** Data curation. **Toby Langstone:** Data curation. **Emily Rothwell:** Data curation. **Claire Atkinson:** Data curation. **Sylvie Pichon:** Writing - original draft. **Fabienne Piras-Douce:** Writing - original draft. **Paul D. Griffiths:** Formal analysis, Writing - original draft. **Matthew B. Reeves:** Formal analysis, Writing - original draft.

Alt-text: Unlabelled box

## 1 Introduction

As with all members of the herpesvirus family, HCMV causes a lifelong, persistent infection in its host. Infection with HCMV is common with sero-prevalence ranging from 45% to 100% [1]. Infection with HCMV is usually asymptomatic, because the immune system in healthy individuals controls the virus. In some settings though, the consequences of the infection or reactivation from latency may be severe, even life threatening (reviewed elsewhere [2]). HCMV viraemia and dissemination is a major cause of end-organ disease development in immunocompromised individuals such as SOT, haematopoietic stem cell transplants (HSC) [3] and late stage HIV patients [4] as well as in fetuses infected in utero [5,6]. In addition, HCMV is associated with adverse outcomes in many patient populations without evidence of end-organ disease [7,8]. The overall socioeconomic burden associated with HCMV is enormous, so a putative vaccine is predicted to be cost-effective, or even cost-saving, and vaccine development has been deemed ‘a top priority’ [9]. Unfortunately, no HCMV vaccine candidate is approaching licensure [10,11].

Arguably, the most successful vaccine studied so far is the recombinant subunit glycoprotein-B with MF59 adjuvant. Phase II clinical trials with seronegative post-partum and adolescent women showed 43–50% reduction in HCMV acquisition among the vaccinated group [12], [13], [14]. Similarly, we reported previously that this vaccine formulation given to seronegative and seropositive patients prior to SOT reduced virological parameters post-transplant. Reduced post-transplant viremia was directly correlated with antibody levels against gB present at the time of transplant suggesting that humoral responses against gB play a role in conferring protection [15]. Our subsequent studies focused on putative mechanisms responsible for the reduction of the virological parameters in these patients revealed little evidence for conventional immune mechanisms of protection in seronegative patients. Specifically, vaccinated seronegative patients displayed little evidence of a neutralizing antibody response against cell-free HCMV in vitro, had minimal effect on the replication of a strain of HCMV engineered to be cell-associated in a viral spread assay and we could not see any evidence of a substantial antibody dependant cellular cytotoxicity-promoting antibody response being generated *de novo* [16]. Similar observations were made in a study of sera from vaccinated seronegative women of child bearing age performed by the Permar lab [17]. Finally, analyses of responses against major antigenic domains of gB following vaccination were variable, and their pattern was distinct when compared with natural infection [16,17]. Therefore, it still remains unclear how the vaccine mediated protection in seronegative recipients. Consequently it was suggested that the vaccine efficacy may have been mediated by non-neutralizing antibody effector functions [16,17].

Here we extend our studies to include serum samples collected post-transplant. In this study we focus on the D+/R- cohort (donor (D) organ from a CMV seropositive individual and recipient (R) CMV seronegative) recruited to the original phase II trial to investigate this. We report that vaccination with gB/MF59 primes the immune system of seronegative recipients to mount larger immunological responses in comparison to recipients of placebo once challenged with the virus, even under conditions of immune suppression during transplantation. Crucially, this boosting of the gB antibody response revealed evidence of gB-specific neutralizing antibodies which were not detected in a prior study of sera post-vaccination but pre-transplant [16]. We propose that this ‘prime-boost’ neutralising antibody response could contribute to the reduced viral load parameters, such as duration of antiviral treatment and incidence of viraemia, seen in this high risk D+R- group, as we reported in the original vaccine trial [15]. More generally, the studies presented here demonstrate the inherent value of studying the immune response post viral challenge as well as post vaccination – an approach made possible by performing studies in our transplant populations in whom the time of infection can be established.

## 2 Materials and methods

### 2.1 Ethics statement

The study was approved by the Research Ethics Committee and all patients whose samples were investigated here gave written informed consent [15].

### 2.2 Population

The population from whom samples have been evaluated and described in this work is the highest risk cohort of seronegative solid organ transplant patients who were enroled in the Phase II randomised and double-blinded placebo controlled cytomegalovirus glycoprotein-B vaccine with MF59 adjuvant trial and received organs from seropositive individuals, described elsewhere [15]. In the original study this cohort represents 11 vaccine recipients and 5 placebo, however, for this study removal of consent for future studies and a patient death mean the data presented is from the remaining 10 vaccine and 4 placebo recipients. No other selection criteria were applied. In short, the vaccine or placebo was given in three doses: at day 0 (baseline), 1 month and 6 months later. Following vaccination, blood samples from patients were obtained consecutively. The patients who subsequently underwent transplantation were followed up for 90 days during which serial blood samples were obtained around days 0, 7, 35, 63, 90. Blood samples after SOT were tested by real-time quantitative PCR (rtqPCR) for cytomegalovirus DNA. CMV PCR was done on a routine basis with an in-house TaqMan (ABI)–based method as described in detail in [18] and [19]. HCMV viraemia was defined as one or more positive HCMV PCR results (cut-off, 200 genomes/mL of whole blood, equivalent to 168 IU/mL). If viraemia higher than 3000 genomes per mL was detected (equivalent to 2520 IU/mL), the patient was treated with antiviral drugs as described in [15]. Exclusion criteria included: pregnancy (a negative pregnancy test was required before each vaccine dose); receipt of blood products (except albumin) in the previous 3 months, and simultaneous multi-organ transplantation [15].

### 2.3 Detection of the total levels of anti-gB antibodies

ELISAs for total gB have been described previously [15,20]. Briefly, sera were diluted in PBS (1:10) and prepared in triplicates. HCMV gB protein (Sanofi Pasteur) was diluted to concentration of 0.75 μg/ml in coating buffer (pH 9.4–9.8). 100μl of the dilution was added to each well of the ELISA plate and incubated overnight at 2–10 °C. All of the following reactions were performed at 37 °C. Reaction wells were rinsed with PBS supplemented with 0.1% Tween-20 (Sigma) then blocked with PBS containing 2% foetal calf serum for 1 h and washed again three times. Following this, serum dilutions were added to the wells and incubated for 1 h. Unbound antibody was removed by washing three times and peroxidase-conjugated secondary antibody (goat-anti-human IgG, Dianova) was added for 1 h. After another three washing steps, 100 µl of tetramethylbenzidine peroxidase substrate was added to each well for 3.5 min, diluted 1:1 in peroxidase substrate solution B (KPL, USA). The reaction was stopped by adding 100 µl of 1 M phosphoric acid to each well. The optical density at 450 nm (OD450) was determined using an Emax microplate reader (Eurofins MWG Operon). Visit 1 (pre-vaccination of seronegative patients) was set as background/baseline.

### 2.4 Qualitative serological analyses (IgM/IgG)

Serological analyses were performed using commercially available line immunoassay kit recomLine CMV IgG and recomLine CMV IgM (Mikrogen Diagnostik) according to manufacturer's instruction. This stripe test contains recombinant antigens to detect IgG and IgM antibodies directed against HCMV from human sera. The antigens included in the test were IE1 (IE1 protein); CM2 (p52 protein, UL44, UL57); p150 (pp150 protein, UL32); p65 (pp65 protein, UL83); gB1 (gB protein, UL55) and gB2 (gB protein, UL55).

### 2.5 Neutralization

To assess sera for neutralizing capacity, HCMV Merlin was pre-incubated with sera or ITC88 antibody [21,22] for 1 h at room temperature, next the sera was used to infect Human foreskin fibroblasts or retinal pigment epithelial cells. The amount of virus used was sufficient to result in an MOI=1 assuming no neutralisation. After 24 h cells were fixed with cold, 100% ethanol (−20 °C) and stained for IE gene expression using anti-IE (Millipore; 1:1000) and goat anti-mouse Alexafluor 568 nm (Life Technologies; 1:1000) at room temperature. Nuclei were counterstained with DAPI (SIGMA; 1:10 000). Percentage infection was enumerated using Hermes WiScan instruments and software. To assess the impact of complement the experimental procedure was identical except that 5% Guinea Pig complement (SIGMA) was added to the sera:virus mix prior to infection.

For the pre-absorption experiment, serum samples (1/10) were pre-incubated with 100 µg/ml of gB vaccine antigen (Sanofi Pasteur) for 1 h at 37 °C prior to incubation with virus.

## 3 Results

### 3.1 Vaccination shortened total duration of viraemia and prevented development of subsequent episodes of viraemia in the D+R- high risk group

**Fig. 1:**
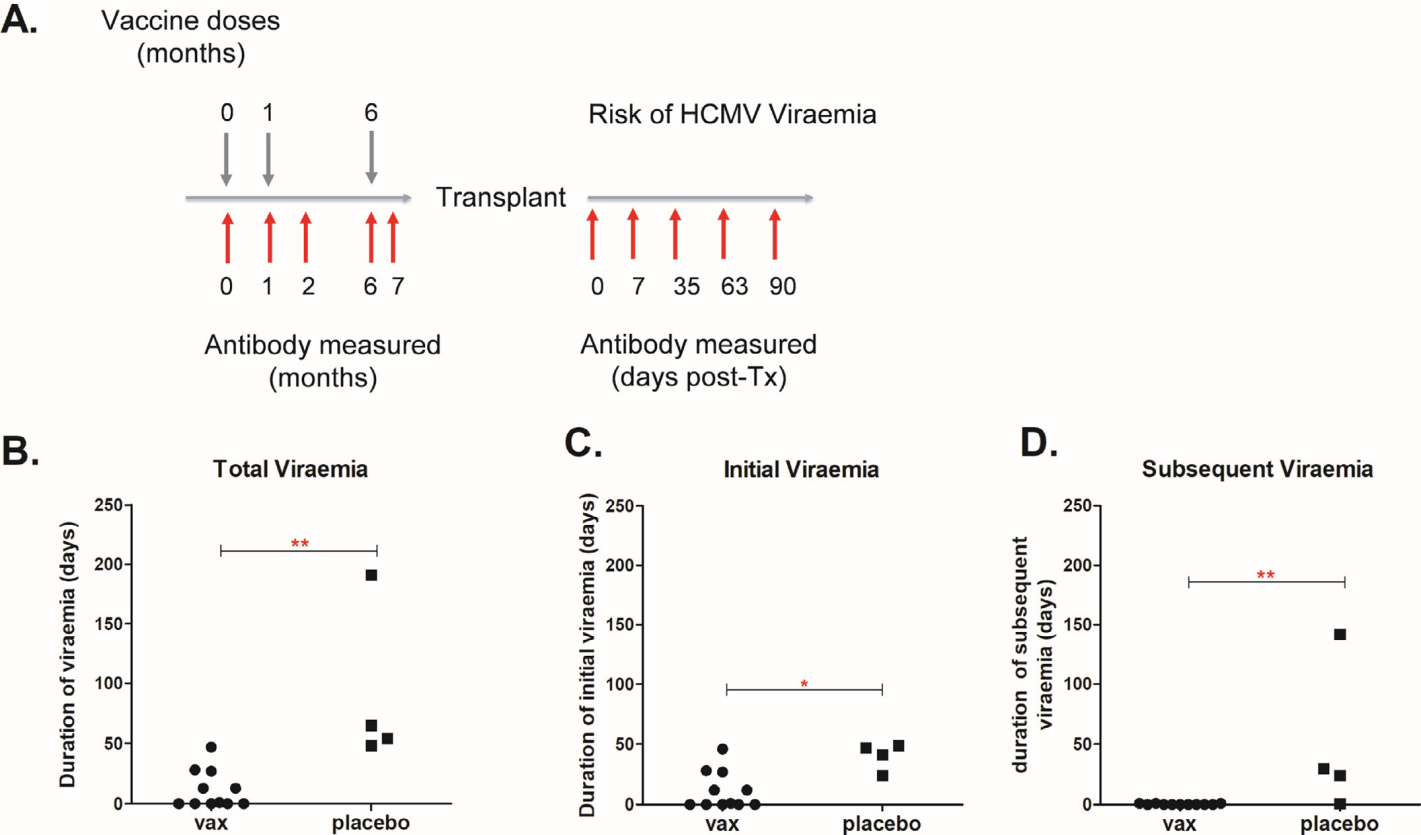
Seronegative vaccine recipients who received organs from seropositive donors are protected from secondary viraemia episodes and have significantly shorter total duration of viraemia post- transplantation in comparison to placebo. A) Schematic representation of the vaccination and sampling schedule. Note time to transplant was variable and not all subjects received 3 doses of vaccine before being called for transplant. B) Total duration of viraemia in vaccine (*n* = 11) vs placebo (*n* = 4) groups of patients. C) Duration of first episode of viraemia in vaccine vs placebo groups of patients. D) Duration of subsequent episodes of viraemia in vaccine vs placebo group of patients. Statistical differences were obtained from Mann Whitney Test; *: *p*<0.05; **: *p*<0.01).

Previously, we reported that gB vaccination reduces the incidence of viraemia in our transplant cohort particularly in our D+R- group. To investigate the clinical impact of gB vaccination in more detail (Fig. 1.A) we analysed a number of virological parameters: total viraemia (duration and occurrence), and duration of initial and subsequent episodes of viraemia in the post-transplant period (Fig. 1). The duration of viraemia was defined as the total number of days on which CMV DNA was detected, including repeated episodes of viraemia in the same patient. A repeat episode of viraemia was defined as the presence of CMV DNA in whole blood detectable following the resolution of a previous episode as documented by two consecutive negative samples. Although the number of available samples was small, these analyses reinforced clear differences between vaccine and placebo recipients. The total duration of viraemia was decreased in the vaccinated group (Fig. 1.B) as well as a reduction in the duration of the first episode of viraemia (Fig. 1.C). Furthermore, no vaccinees experienced second episodes of viraemia (0/11) in comparison to 75% of placebo patients (3/4) who had more than one episode of viraemia (Fig. 1.D).

### 3.2 A rapid increase in gB antibody levels was observed post-transplant in seronegative vaccinees

**Fig. 2:**
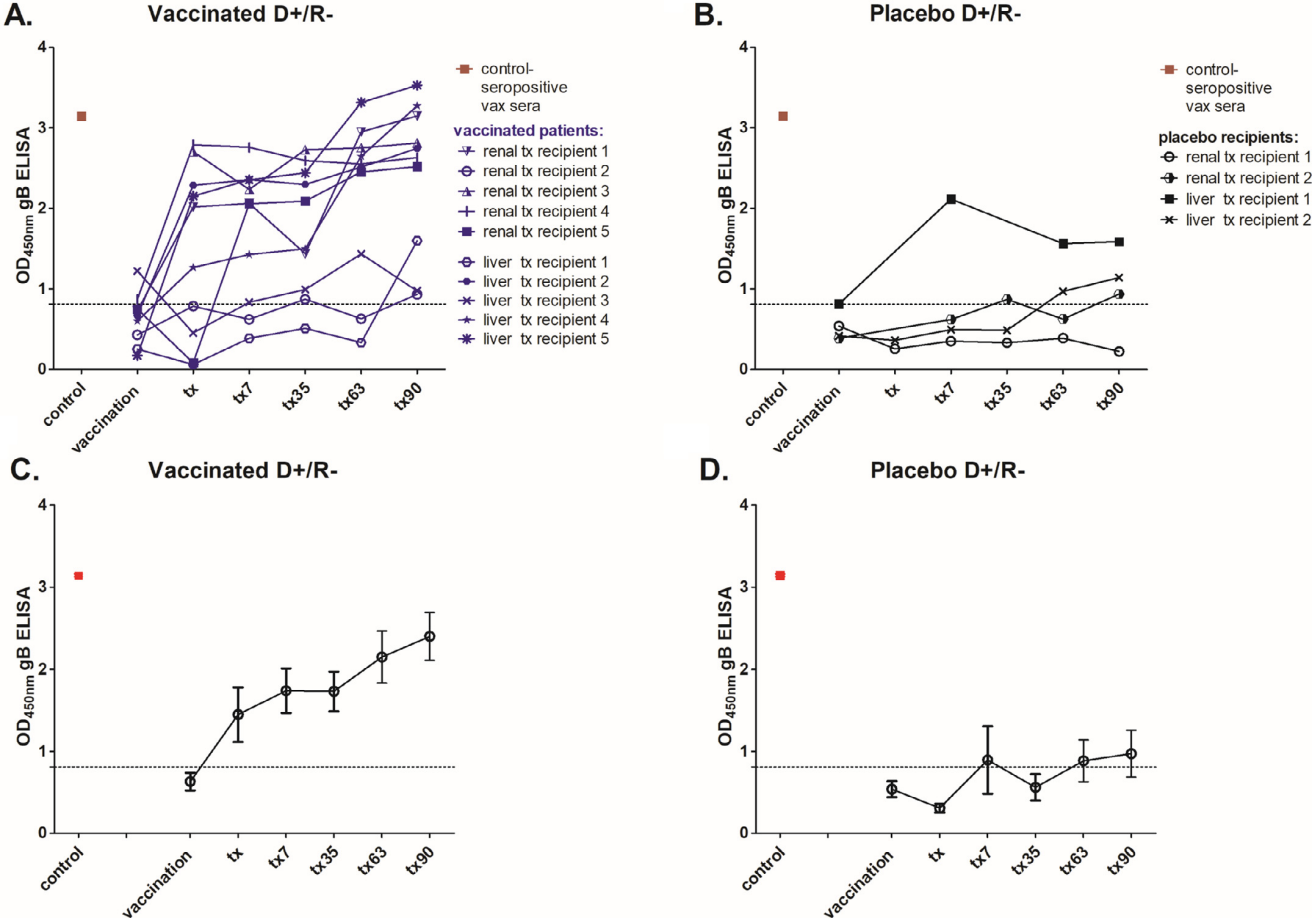
Seronegative patients who received vaccination prior to transplantation are primed to mount faster and more robust anti-gB antibody responses once challenged with the virus in comparison to delayed responses in placebo recipients. Total anti-gB antibody levels were measured by standard ELISA and are shown as follows: A) for individual vaccinees (*n* = 10) and B) placebo (*n* = 4) recipients; C-D) The average anti-gB antibody responses are presented for vaccine (C) placebo patients (D): Serum samples were collected at the time of vaccination (baseline, dotted line), time of transplantation and on days: 7, 35, 63 and 90 post-transplant. Where an individual point (in A & B) is missing a sample was not available for that timepoint for analysis. Combined sera from healthy seropositive donors (*n* = 6) was used as a positive control. Dashed line represents the cut off and was informed by control sera taken from healthy seronegative individuals (*n* = 6).

**Fig. 3:**
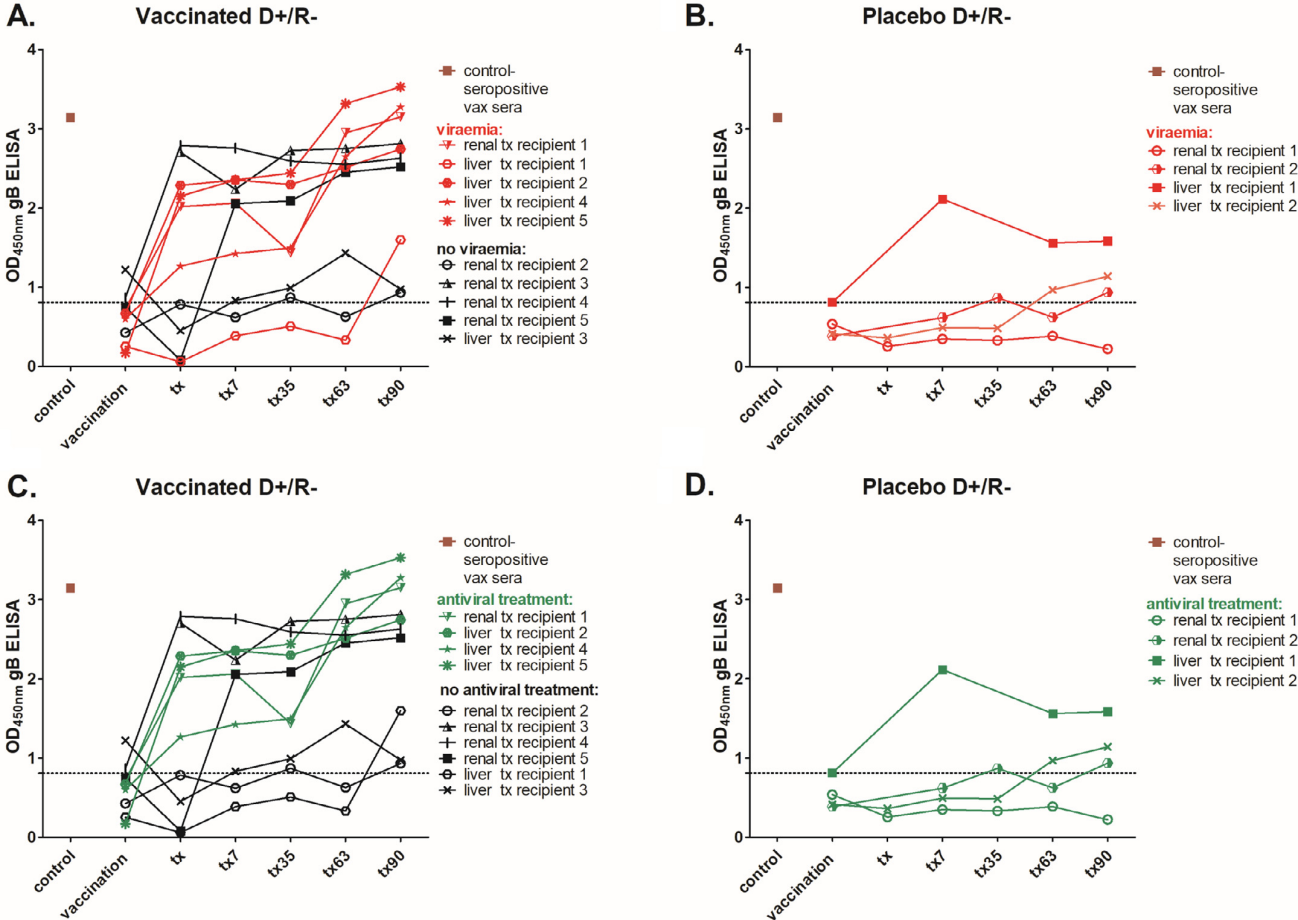
A prompt post-transplant increase in antibody levels against glycoprotein B is not explained by either low level or high level viraemia. Total anti-gB antibody levels were measured by standard ELISA and are shown for individual patients. A-B) Patients were stratified for viraemia (red) versus non-viraemia (black) in vaccinated (A; *n* = 10) and placebo recipients (*n* = 4; B). C-D) Alternatively, vaccine (C) or placebo (D) patients were stratified by requirement for antiviral treatment (viraemia exceeding 3000 genome copies/ml) and are depicted in green: Serum samples were collected at the time of vaccination time of transplantation and on days: 7, 35, 63 and 90 post-transplant. Dashed line represents the cut off and was informed by control sera taken from healthy seronegative individuals (*n* = 6). (For interpretation of the references to color in this figure legend, the reader is referred to the web version of this article.)

Our original analyses had focused on the patient sera collected during the pre-transplant immunisation phase in order to identify evidence for strong correlates of protection pre-transplant. However, we hypothesised that important elements of the humoral response in the vaccinees may only reveal themselves upon challenge with the virus, so we investigated immunological parameters in the post-transplant sera. Firstly, total level of anti-gB IgG antibodies was measured by standard enzyme immunoassay to investigate the magnitude of humoral responses in the three month long post-transplantation surveillance period. A caveat is that the number of patients in this high risk D+R- setting who proceeded to transplant was low which limited analyses. In the placebo patients (*n* = 4) three had gB antibody levels below the negative cut off baseline throughout most of their post-transplant period and only one placebo patient developed detectable anti-gB antibodies early post-transplant (Fig. 2.B, D). This individual along with the other three placebo patients experienced viraemia (Fig. 3.B). Generally, we observed slow and moderate increase of the antibody responses against gB in placebo recipients, which became detectable at the end of this 90 day surveillance period (Fig. 2.B, D). Interestingly, the kinetics of the anti-gB antibody responses in our vaccinees (*n* = 10) in this high risk D+R- group was different. On average, vaccinated individuals produced detectable responses against gB more quickly than did placebo recipients once challenged with the virus. Indeed, increased IgG responses were detectable within 7 days of transplant in the majority of vaccinees (Fig. 2.A, C). However, the magnitude of the gB response post-transplant was not necessarily a clear indicator of outcome alone (Fig. 3). Five vaccinees with high gB responses post transplant experienced viraemia of which four required antiviral treatment (Fig. 3A,C).

### 3.3 The rapid increase in gB responses in vaccinated individuals post-transplant is linked to a boost of pre-existing anti-gB responses

The rapid increase in gB IgG levels suggested that challenge with HCMV at the time of transplant was either promoting new antibody responses or was boosting pre-existing antibody responses that had been generated by vaccination with gB prior to transplant. Essentially, the vaccine recipients were gB-seropositive but HCMV seronegative at the time of transplant. A lack of gB response in the placebo control group argued against the induction of a new antibody response to infection and thus the composition of the humoral response post-transplant was investigated further. To look for evidence of new responses to infection IgM antibodies against major HCMV antigens in the post-transplant sera from D+R- group were measured by enzyme immunoassay. No IgM responses were detected against HCMV antigens in placebo recipients at days 7 or 35 post-transplantation (Table S1A). Similarly, no IgM responses were detected in the majority of vaccinated D+R- patients at days 7 and 35 post transplantation. However, two vaccine patients did have detectable IgM responses to HCMV antigens other than gB (Table S1B).

### 3.4 Neutralizing antibody responses were detected in sera from seronegative vaccine recipients in the post-transplant period

**Fig. 4:**
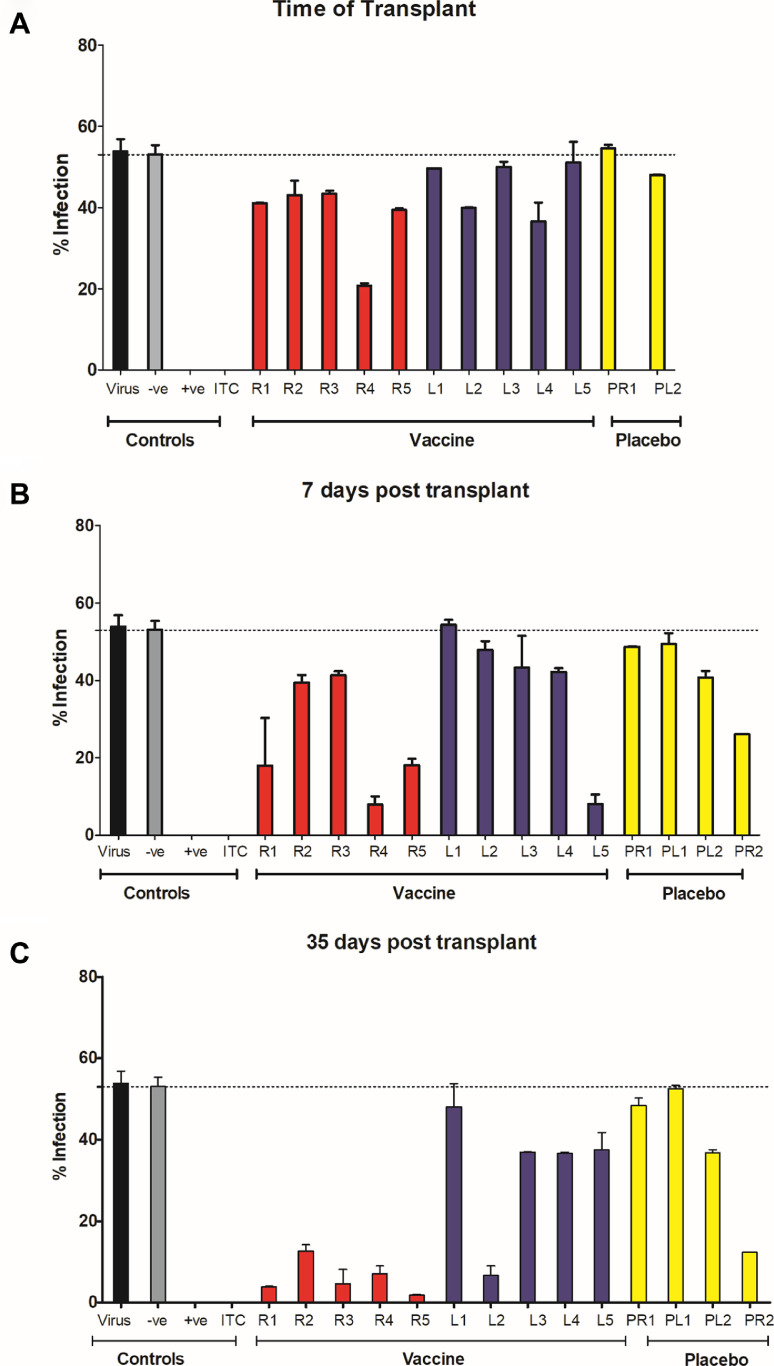

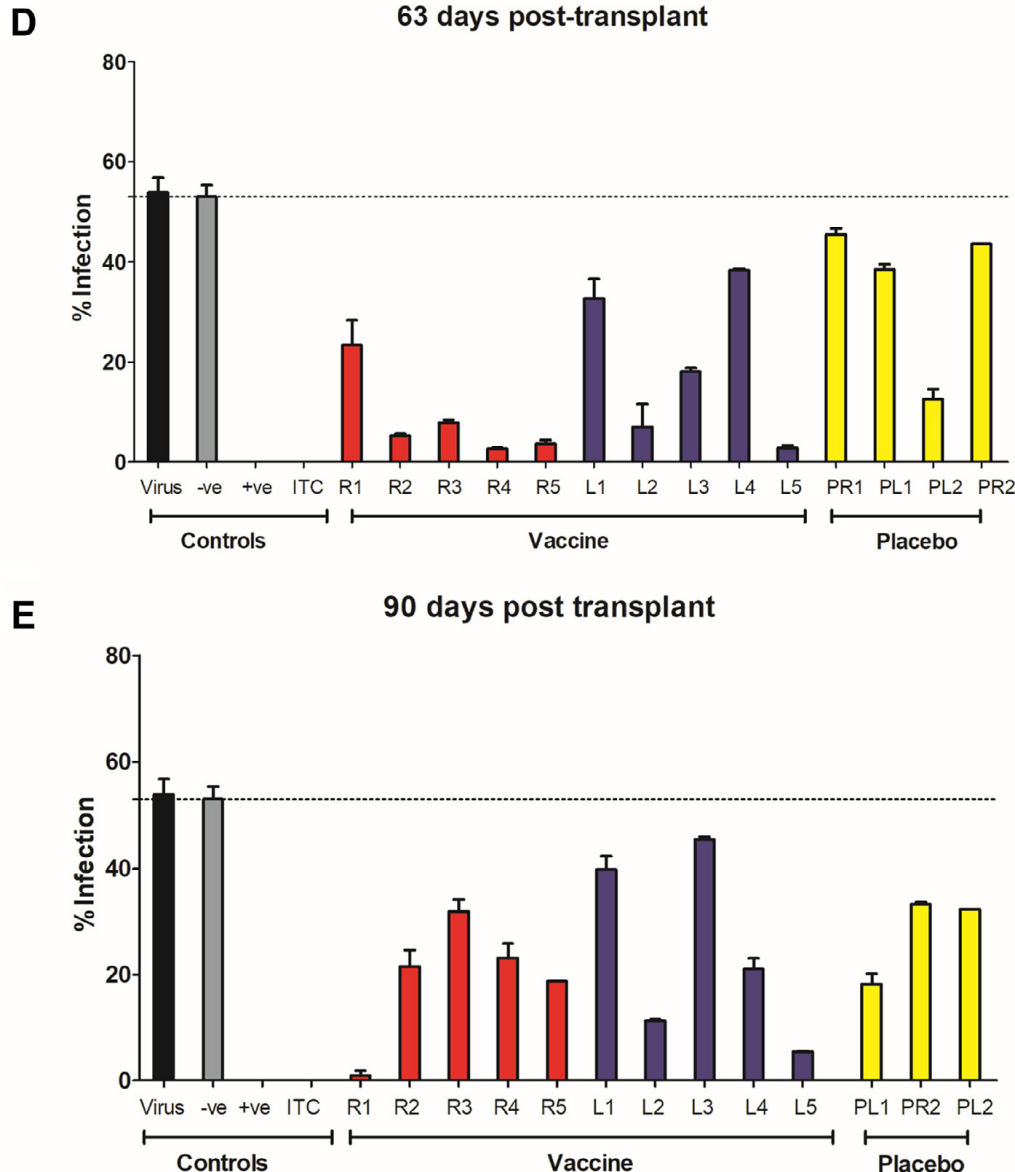
Neutralizing antibody responses in post-transplant serum samples from vaccinated individuals appear faster than in placebo recipients. HCMV (Merlin strain) was incubated with neutralizing antibody ITC88 (ITC) or sera (1:10) from seronegative patients from the phase II trial. For controls, pooled serum from healthy seropositive (+ve) or seronegative (-ve) donors were used (*n* = 10). Following incubation, the antibody:virus mixes were used to inoculate HFFs in vitro (MOI=1). Infection was measured by IE staining and the proportion of infected cells calculated by counterstaining nuclei with DAPI. Sera was analysed from vaccine recipients receiving a kidney (R1-R5; red) or liver (L1-L5; blue), or placebo recipients (yellow) receiving a kidney (PR1 & 2) or liver (LR1 & 2). The average percentage infection following incubation with post-transplant serum is shown for each individual A) at the day of transplantation B) 7 days post transplantation, C) 35 days post transplantation, D) 63 days post transplantation and E) 90 days post transplantation. (For interpretation of the references to color in this figure legend, the reader is referred to the web version of this article.)

**Fig. 5:**
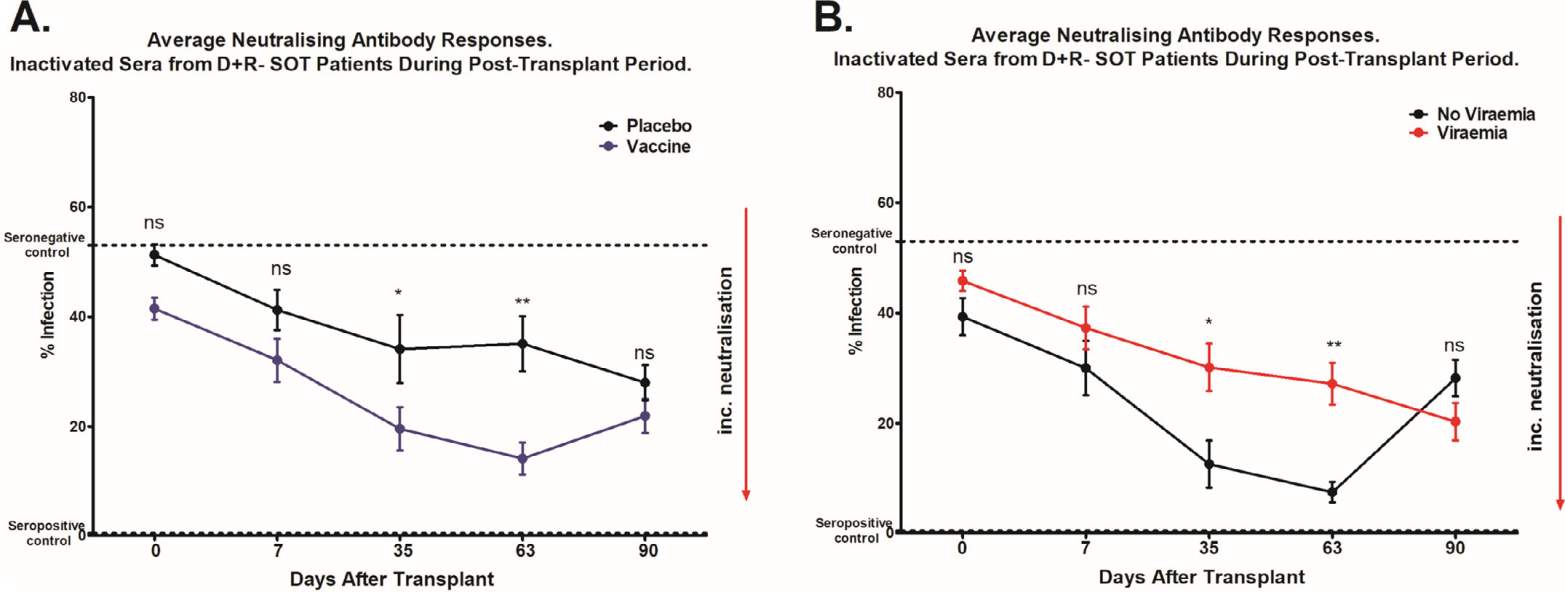
Sera collected during post-transplant period show superior neutralization capacities of vaccinated seronegative transplant patients in comparison to placebo. Strain Merlin was incubated with sera from seronegative patients, or pooled patient seropositive or seronegative sera as controls (*n* = 6) and used to inoculate HFFs in vitro (MOI=1). Infection was measured by IE staining and the proportion of infected cells calculated by counterstaining nuclei with DAPI. A) The average percentage infection of all the vaccinated and non-vaccinated heat inactivated patient sera; vaccine recipients are shown in blue (lower line) and those who received placebo are shown in black (upper line) B) The average percentage infection of all the vaccinated and non-vaccinated heat inactivated patient sera; patients who developed viraemia post-transplant are shown in red (upper line) and those who did not are depicted in black (lower line). Serum samples were collected at the time of transplantation and on days: 7, 35, 63 and 90 post-transplant. A comparison of neutralisation at each timepoint was performed by Mann U Whitney Test. *p<0.05, **p<0.01 and ns=non-significant difference.

The data thus far argued for a vaccine dependant boost in gB antibody levels post challenge with HCMV (i.e. post-transplant). The key question, however, was whether this boost in gB antibody levels may reveal a hitherto undetectable response in the pre-transplant sera that could explain vaccine efficacy. Thus we asked whether the increase in gB IgG levels early post-transplant revealed evidence of neutralizing activity previously undetectable in the pre-transplant sera samples [16]. Neutralisation was scored by the ability of the sera to prevent lytic infection of fibroblasts when pre-incubated with the virus prior to infection. Experimental conditions were established whereby seronegative pooled patient serum had no impact on virus infectivity whilst the well characterised neutralizing antibody ITC88 showed almost complete neutralization of infection (Fig. 4 and Fig. 5).

Interestingly, the analysis of sera from seronegative vaccine recipients in the post-transplant period showed evidence of neutralizing antibody activity as soon as 7 days post-transplant and the majority of those analysed had antibodies that were able to neutralize infection at day 35 post-transplant (Fig. 4A–C and 5A). The neutralising activity observed in the sera taken from the vaccinees post transplant was largely complement independant (Fig. S3). Furthermore, the sera also neutralised infection of epithelial cells – consistent with the role gB plays in the entry of HCMV into all cell types (Fig. S4). The neutralising activity observed in vaccinees’ sera was in contrast, recipients of placebo whose sera exhibited little or no neutralizing activity at those early time points post-transplant (Figs. 4A–C and 5A). However, in analyses of sera taken at later time points post-transplant, evidence of a neutralizing antibody response developing in these placebo individuals was also observed. Indeed, by day 90 post-transplant, similar levels of neutralization were present in both groups (Figs. 4.D,E and 5A).

Next, we sought to investigate whether the presence of neutralizing antibodies early post-transplant in our vaccinees correlated with occurrence of viraemia, duration of viraemia or duration of antiviral therapy. Although sample size was limited, the analyses showed that, on average, sera from patients who developed viraemia had significantly lower levels of neutralizing activity at the early time-points post transplantation (Fig. 5.B). Moreover increased neutralizing activity in the sera tended to correlate with reduced duration of viraemia (Fig S1.A–C) and length of antiviral therapy (Fig. S2.A–C), although the associations were not statistically significant.

**Fig. 6:**
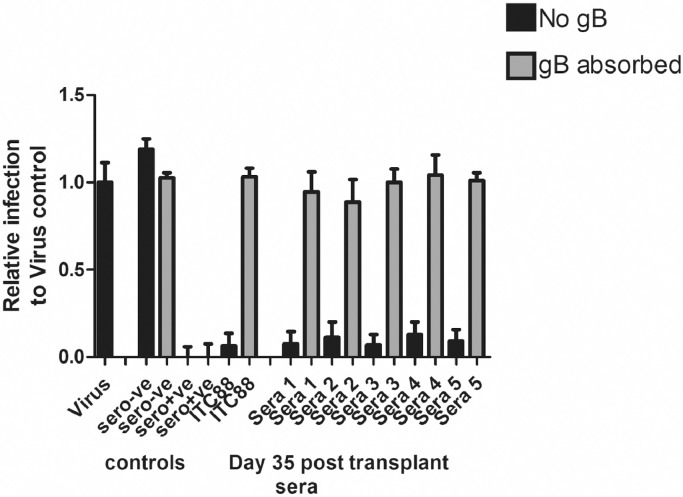
The neutralising activity in the seronegative vaccine sera is inhibited following pre-absorption with recombinant gB. Sera from healthy seronegative (sero-ve) and seropositive (sero+ve) individuals and sera from vaccinated seronegative SOT recipients collected 35 days post-transplant were diluted 1/10 and incubated with the virus prior to infection of the fibroblasts for 1 h (black bars). Alternatively, virus was incubated with ITC88 (100ug/ml) for 1 h prior to infection (ITC88 black bar). Additionally, each serum and ITC88 were also pre-incubated with the vaccine gB antigen prior to incubation with the virus in parallel (grey bars). Infection was measured by IE staining and the proportion of infected cells calculated by counterstaining nuclei with DAPI.

Finally, to test if the neutralizing activity observed at early times post infection in our vaccinees was due to anti-gB responses we hypothesised that pre-absorption of sera from seronegative vaccinated individuals (collected at day 35 post-transplant), with vaccine antigen recombinant gB should reverse the neutralisation. To do this, we selected the serum samples with the highest neutralising response (Fig. 4) and tested them in a gB pre-absorption assay. Consistent with the hypothesis, the neutralising activity detected in our vaccinee sera at d35 was removed by the addition of recombinant gB (Fig. 6). Interestingly, the same approach in control seropositive donor sera failed to have any appreciable impact on its’ neutralising activity (Fig. 6).

## 4 Discussion

Despite important studies spanning several decades of HCMV vaccine research, there is still no HCMV vaccine licensed for clinical practice. An important feature of HCMV that may be impacting on progress is the natural history which, arguably, is much more complex than viruses that have previously been controlled by immunization to prevent primary infection [23]. For instance, the threat from HCMV can manifest from primary infection and also reactivation from latency as well as re-infection (i.e. the ability of HCMV to infect hosts despite prior immunity) [24,25].

A major correlate of protection of many successful vaccines has been neutralizing antibodies [23]. However, reactivation and re-infection with HCMV likely occurs in individuals who have already formed endogenous neutralizing antibody responses suggesting that these antibodies are not sterilizing although they may contribute to natural control in the host [26]. Indeed, our analyses of humoral responses in sera collected pre-transplant in vaccinated seronegative patients showed poor neutralizing capacities as well as very little functional activity in our assays. Therefore, we concluded that the effectiveness of the gB vaccine in this group of individuals could not be explained by the presence of neutralizing antibodies and, consequently, might be imparted by a novel mechanism that would be revealed by more sophisticated analyses [16].

To build on this insight, we took advantage of the unique nature of the transplant model – which effectively is a human challenge model with a defined time-point of HCMV infection post vaccination [27]. The pharmacodynamic assessment of the post-transplant sera of seronegative vaccine and placebo recipients from the D+R- high risk group offers a potential unbiased, open-ended way to discovery as long as a robust biomarker is available as the read out of successful control of the disease process [18,19]. In our patient cohort, post-transplant viral load is an accepted natural biomarker to allow this approach to be undertaken and various parameters of viraemia are sufficiently robust to be used as endpoints in clinical trials [28].

Although the sample sizes were small, the data herein show that vaccinated patients did not experience subsequent episodes of viraemia. This suggests that these patients gain control of the virus much quicker post-transplant. We hypothesized that the control was driven by adaptive immune responses against gB and, particularly in the early times post-transplant, could be linked with the humoral response given that these patients are T cell immune-suppressed. Consistent with this, a rapid increase in the level of anti-gB antibodies post-transplant was observed in some vaccine recipients as early as day 7 post transplant. In comparison a gB antibody response was only detectable much later (>35 days) in the placebo group. One possible interpretation is that vaccinated individuals were primed to generate a rapid response from memory when they encountered HCMV from the donor organ. However, it was important to note that it was not simply the magnitude of the gB response that was important post-transplant. This is in contrast to pre-transplant where the gB antibody titre in response to vaccination was a clear correlate of protection against duration of viraemia [15]. The most likely reason for this difference is the additional variable of viral infection at the time of transplant. Although these individuals are immune-suppressed they are still capable of forming some level of immune response. Furthermore, viral strain and the inoculum titre could all contribute to more complex responses post transplant.

The rapid onset of IgG gB and a general lack of IgM responses against gB (or other viral epitopes) is consistent with boosting of a pre-existing response in the primed B cells of these T-cell suppressed hosts. Indeed, the comparative delay seen in the placebo group would argue that immune-suppressed patients cannot make novel antibody responses against HCMV during the early stages post-transplant (however, we cannot rule out that in our sampling between Day 0 and day 7 post transplant we may have missed an IgM response). Here it is important to re-iterate that no statistical correlation between total antibody levels post-transplant and outcome could be made. For example, some vaccinated individuals had evidence of less boosting post-transplant but were protected. The possibility that pre-formed antibodies may have prevented transmission of virus from the donor (which would prevent downstream viraemia and a boosting effect) should be examined in future vaccine studies with larger patient cohorts than available here.

Perhaps the most important aspect of this study is the suggestion that vaccine can induce a neutralizing antibody response against gB but that this was only revealed post-transplant. Evidence of neutralizing activity was observed as early as 7 days post-transplant in some vaccinees, with the majority of them having antibodies that were able to neutralize infection at day 35 post-transplant. An important aspect of this was that the neutralizing antibody response in the vaccinees was negated using the gB antigen. In contrast, the gB antigen did not reverse the neutralizing activity of sera from healthy seropositive donors. Essentially, the vaccinees have only generated antibody responses against gB and thus the neutralizing response is dominated by gB responses at those early times post transplant. However, it is likely that if these individuals seroconvert the dominance of gB antibodies in the repertoire will be reduced. The gB antibody phenotype of the response seen in our vaccinees at 7–35 days post transplant rarely happened in the recipients of placebo although, over time, they did also develop neutralizing antibody responses. The development of these responses in our placebo controls were possibly linked with a reduction of immune-suppression over time and probably included responses targeted against multiple antigens on HCMV. However, in the context of vaccination, despite a very limited sample size, the analyses suggest that neutralization may be inversely correlated with the duration of viraemia- a possibility that should be examined in future larger clinical trials.

This in itself raises some important questions. The role of neutralizing antibodies for the activity of the gB vaccine has been debated. In two separate analyses, vaccination did not induce measurable levels of neutralizing antibody responses against gB [16,17]. However, analyses of sera taken from a phase I study of the gB/MF59 vaccine revealed potent complement dependant neutralizing activity in gB antibody repertoire of vaccinated individuals [29]. It is likely that none of these studies are contradictory. Indeed, we now report evidence of neutralizing activity post transplant which we believe to be the result of a boosted IgG response against gB. Thus, if the hypothesis is correct, that neutralizing response was present pre-transplant, but below the level of detection in our assays. Hypothetically, if it is the challenge with the virus that is revealing this then it is possible that time of sampling in the other studies of women of child bearing age is important. Unlike in this study, where the time of viral challenge is known, it represents a potentially measurable factor in those studies that could be incorporated into the design of future vaccine studies. This could help reconcile further the apparent differences reported by different groups – including ourselves.

Taken together, despite the severe limitations of sample size that have limited many of the follow-up analyses presented here, the data suggest that vaccine recipients were immunologically primed by vaccination and that transplant, and thus challenge with HCMV, induced functional antibody responses that had been primed by vaccination. A correlate of this was that these vaccine recipients inhibited viral infection more effectively in comparison to the placebo group. We suggest that future studies on HCMV vaccines in SOT should include analysis of the sera (and lymphocytes) collected post transplantation to allow comprehensive follow up analyses to further define the mechanistic basis for the protective effect seen with the gB/MF59 vaccine and to begin to assess the impact of viral infection on the immune response generated by vaccination against HCMV in seronegative individuals.

## Declaration of Competing Interest

S.P. and F.D.P. are employees of Sanofi Pasteur. All other authors declare no conflicts of interest.

## Appendix Supplementary materials

Image, application 1
