# Supplementary material for: Seronegative patients vaccinated with cytomegalovirus gB-MF59 vaccine have evidence of neutralising antibody responses against gB early post-transplantation
Source: eBioMedicine. 2019 Nov 15;50:45–54. doi: 10.1016/j.ebiom.2019.11.005 (PMC6921368; doi:10.1016/j.ebiom.2019.11.005)
Supplement: Supplementary file 1 [file mmc1.docx]

**Supplementary Figure Legends & Tables:**

|  | **placebo recipients** | **viraemia** | **Day** | **IE1 (UL123)** | **CM2 (UL44, UL57)** | **p150 (UL32)** | **p65 (UL83)** | **gB1 (UL55)** | **gB2(UL55)** |
| --- | --- | --- | --- | --- | --- | --- | --- | --- | --- |
| IgM | Renal tx 1 | Yes | 7 | no | no | no | no | no | no |
|  |  |  | 35 | no | no | no | no | no | no |
| IgM | Renal tx 2 | Yes | 7 | no | no | no | no | no | no |
|  |  |  | 35 | no | no | no | no | no | no |
| IgM | Liver tx 1 | Yes | 7 | no | no | no | no | no | no |
|  |  |  | 35 | no | no | no | no | no | no |
| IgM | Liver tx 2 | yes | 7 | no | no | no | no | no | no |
|  |  |  | 35 | no | no | no | no | no | no |

**Table S1A)**

**Table S1B)**

|  | **vaccine recipients** | **viraemia** | **Day** | **IE1 (UL123)** | **CM2 (UL44, UL57)** | **p150 (UL32)** | **p65 (UL83)** | **gB1 (UL55)** | **gB2 (UL55)** |
| --- | --- | --- | --- | --- | --- | --- | --- | --- | --- |
| IgM | Liver tx 1 | Yes | 7 | no | no | no | no | no | no |
|  |  |  | 35 | no | no | no | no | no | no |
| IgM | Liver tx 2 | Yes | 7 | no | no | no | no | no | no |
|  |  |  | 35 | no | no | no | no | no | no |
| IgM | Liver tx 3 | No | 7 | no | no | no | no | no | no |
|  |  |  | 35 | no | no | no | no | no | no |
| IgM | Liver tx 4 | Yes | 7 | no | no | no | no | no | no |
|  |  |  | 35 | no | no | no | no | no | no |
| IgM | Liver tx 5 | Yes | 7 | no | no | no | no | no | no |
|  |  |  | 35 | no | no | no | no | no | no |
| **IgM** | **Renal tx 1** | **Yes** | **7** | **no** | **yes** | **no** | **yes** | **no** | **no** |
|  |  |  | **35** | **no** | **no** | **no** | **no** | **no** | **no** |
| **IgM** | **Renal tx 2** | **No** | **7** | **no** | **yes** | **no** | **no** | **no** | **no** |
|  |  |  | **35** | **no** | **yes** | **no** | **no** | **no** | **no** |
| IgM | Renal tx 3 | No | 7 | no | no | no | no | no | no |
|  |  |  | 35 | no | no | no | no | no | no |
| IgM | Renal tx 4 | No | 7 | no | no | no | no | no | no |
|  |  |  | 35 | no | no | no | no | no | no |
| IgM | Renal tx 5 | no | 7 | no | no | no | no | no | no |
|  |  |  | 35 | no | no | no | no | no | no |
|  |  |  |  |  |  |  |  |  |  |

**Tab S1. Serological analyses show very poor/undetectable IgM responses against HCMV in D+R- group during the post-transplant period.**

A) placebo recipients (n=4) B) vaccine recipients (n=10).

**Supplementary figure Legends**

**Fig S1. Non-significant trend associating neutralization inversely with the duration of viraemia post-transplant.**

The capacity of neutralization measured as a decrease in the percentage of infectivity in an in vitro neutralisation assay with patient sera is shown for each placebo (dots) and vaccine (triangles) individual from D+R- group plotted against the duration of viraemia: A); B); C); Neutralization capacity of the sera was measured at different time-points: day of transplantation A), 7days post-transplantation B), and 35 days post-transplant C).

**Fig S2. Non-significant trend associating neutralization inversely with the duration of antiviral therapy post-transplant.**

The capacity of neutralization as measured as a decrease in the percentage infectivity of Merlin in an in vitro neutralisation assay with patient sera is shown for each placebo (dots) and vaccine (triangles) individual from the D+R- group plotted against the total length of antiviral therapy: A); B); C). Neutralization capacity of the sera was measured at different time-points: day of transplantation A), 7days post-transplantation B), and 35 days post-transplant C).

**Fig S3. Neutralisation is not enhanced by complement**

Sera from healthy seronegative (sero-ve) and seropositive (sero+ve) individuals and sera from vaccinated seronegative SOT recipients collected 7 (A,B) or 35 (C,D) days post-transplant were heat inactivated and then diluted 1/10 and incubated with the virus prior to infection of the fibroblasts for 1h in an absence (black bar) or presence of 5% guinea pig complement (grey bar). Infection was measured by IE staining and the proportion of infected cells calculated by counterstaining nuclei with DAPI.

**Fig S4. Post transplant sera from vaccinees neutralises infection of epithelial cells**

Sera from healthy seropositive (sero+ve) individuals and sera from vaccinated seronegative SOT recipients collected 7 (A) or 35 (B) days post-transplant were heat inactivated and then diluted 1/10 and incubated with the virus prior to infection of the epithelial cells for 1h. Infection was measured by IE staining and the proportion of infected cells calculated by counterstaining nuclei with DAPI. Infection was then scored relative to virus infection control (Virus)
